# Supplementary material for: Topologically protected Dirac plasmons in a graphene superlattice
Source: Nat Commun. 2017 Nov 1;8:1243. doi: 10.1038/s41467-017-01205-z (PMC5665919; doi:10.1038/s41467-017-01205-z)
Supplement: Supplementary file 1 — Supplementary Information [file 41467_2017_1205_MOESM1_ESM.pdf]

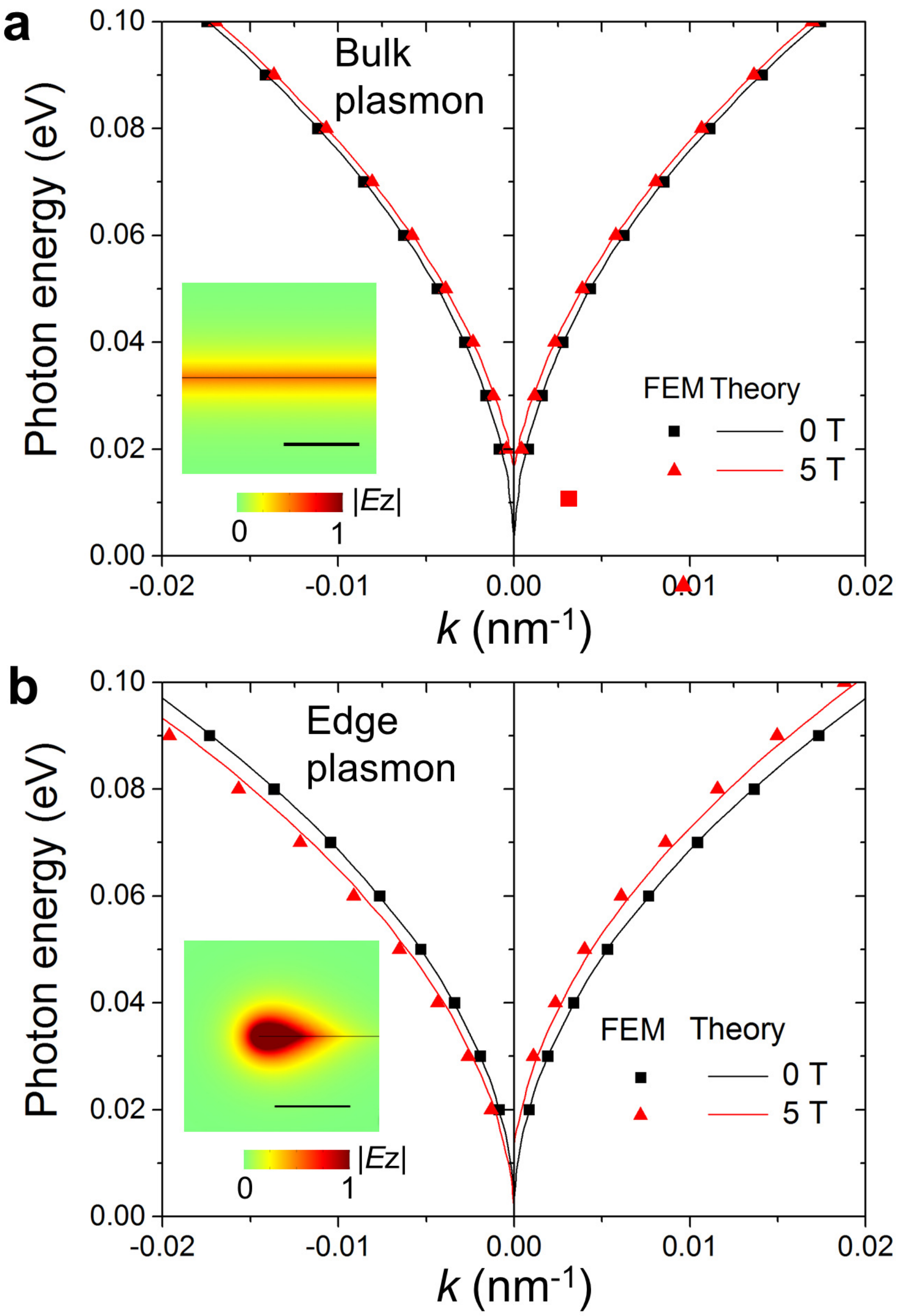

Supplementary Figure 1. Dispersion relations for graphene magnetoplasmons. (**a-b**) Dispersion relations for bulk (**a**) and edge (**b**) plasmons on freestanding graphene. Simulation and theoretical results are shown for zero magnetic field (red) and for a magnetic field of 5 T (black). Insect: distributions of the electric-field component along the propagation direction. Scale bars: 200 nm.

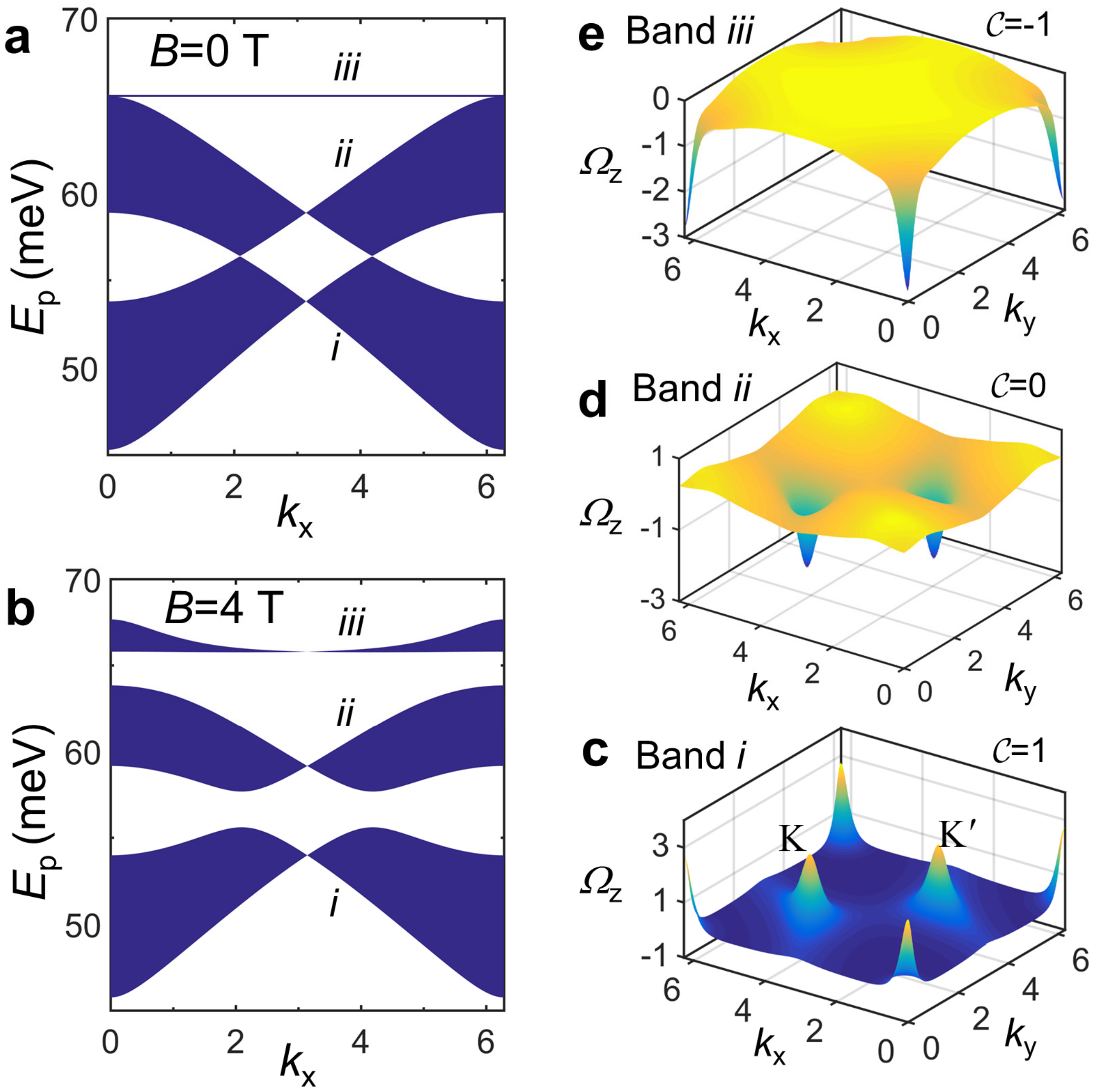

Supplementary Figure 2. Berry curvatures of relevant bands. (**a-b**) Projected band diagrams along the zigzag direction for magnetic fields *B*=0 (**a**) and *B*=4 T (**b**). (**c-e**) Distribution of Berry curvatures in the first Brillouin zone for band *i*, *ii*, and *iii* in **b**. The integral of the Berry curvatures yields the corresponding Chern numbers shown here as labels.

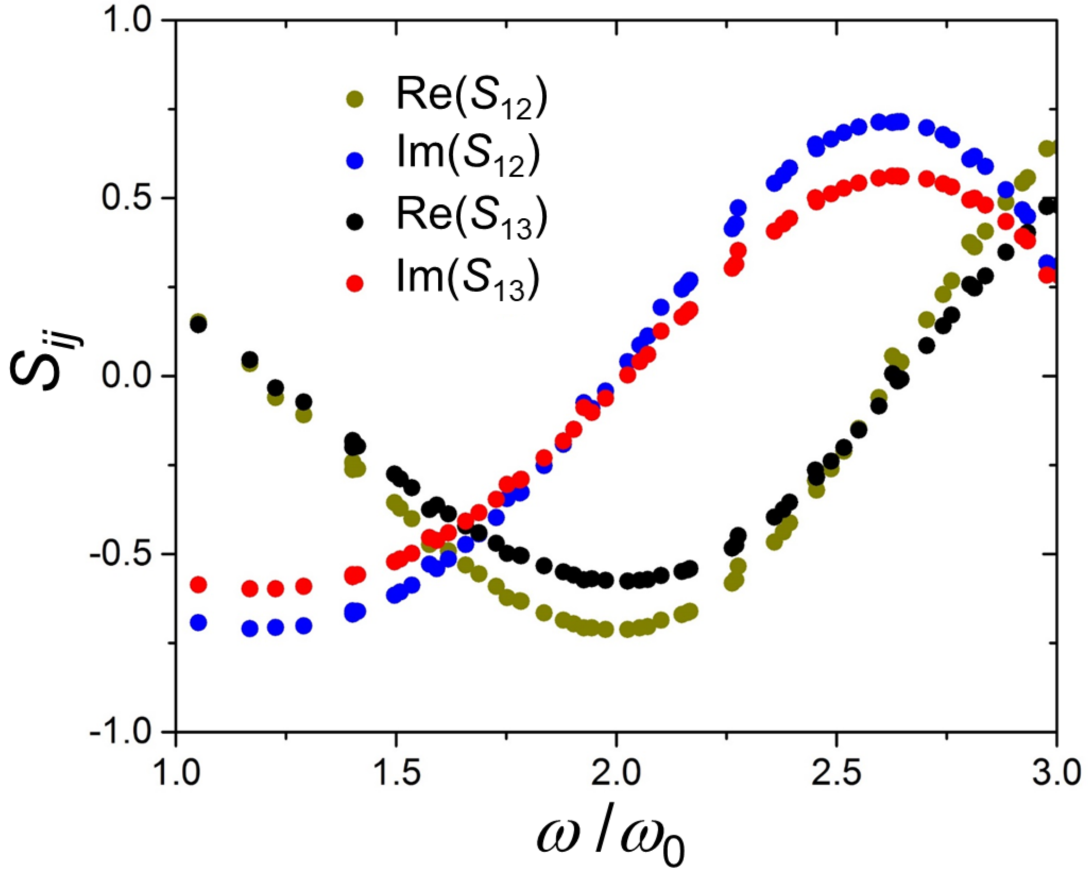

Supplementary Figure 3. Electrostatic scaling of the graphene superlattice. We show the scattering coefficients of graphene nanoribbon junctions (with $2L=3W$ as in the superlattice of Figs. 2 and 3) for all combinations of the parameters B=0.8, 2 T, E_F =0.2, 0.3 eV, and ε_2=1, 2.1, as a function of ω/ω_0. The size is adjusted to ensure that ω_c/ω_0 is the same as in the right panel of Fig. 4d for all calculations.

Supplementary Note 1. Magnetoplasmon modes on graphene nanoribbon.

A layer of graphene supports bulk plasmons (Supplementary Fig. 1a) and more localized edge plasmons at the boundary (Supplementary Fig. 1b). The dispersion relations of bulk plasmons $\omega_{\mathrm{B}}(k)$ (determined by $k = 2i\omega\varepsilon_0/\sigma_0(\omega)$) and edge plasmons are correlated: $\omega_{\mathrm{E}}(k) = (2/3)^{1/2}\,\omega_{\mathrm{B}}(k)$ [1], where the prefactor implies the localization of the edge plasmons. With magnetic field, the dispersion of the bulk plasmons is shifted to $\omega_{\mathrm{B}}'^2 = \omega_{\mathrm{B}}^2 - \omega_{\mathrm{c}}^2$, where ω_{c} is the cyclotron frequency defined in the main text, while the edge plasmons become $\omega'_{\mathrm{E},\pm} = \sqrt{2}\left[\left(3\omega_{\mathrm{B}}^2 + \omega_{\mathrm{c}}^2\right) \pm \omega_{\mathrm{c}}\right]/3$ [2-5], where the signs before ω_{c} correspond to the direction of the magnetic field normal to the carbon plane. Because of the symmetry of the system, for the edge magnetoplasmon, reversing the direction of the magnetic field is identical to reversing the direction of the wave vector, $\omega'_{\mathrm{E},\pm}(q) = \omega'_{\mathrm{E}}(\pm q)$. Therefore, the edge magnetoplasmon is nonreciprocal: $\omega'_{\mathrm{E}}(+q) \neq \omega'_{\mathrm{E}}(-q)$, in contrast to the bulk magnetoplasmon, as shown in Supplementary Fig. 1.

The two lowest-order modes of the nanoribbon are bonding and antibonding combinations of the two edge charge pileups on opposite boundaries of the nanoribbon (inset in Fig. 1b). For a graphene ribbon under a magnetic field, the edge modes on the two boundaries propagating toward the same direction have different

wave vectors. The combinations of these two edge modes are analogous to the asymmetric supermodes of two nonidentical coupled waveguides, leading to the results shown in Fig. 1b.

Supplementary Note 2. Results of Berry curvatures

As discussed in the main text, the Chern number of the first band below the gap determines the topological edge mode in the gap, because a lower trivial bandgap exists. The Berry curvature of this band (denoted as band *i* in Supplementary Fig. 2a,b) shown in Supplementary Fig. 2b reveals the mechanism of the nonzero Chern number of this band. Apart from the peak at the corner of the first Brillouin zone due to the narrowing of the lower bandgap, it also displays two peaks of finite height at the K and K' points when a bandgap is opened at the Dirac points. For the bandgap opened by T-symmetry breaking as in our study, the two peaks in the Berry curvature of band *i* both have positive signs, as shown in Supplementary Fig. 2c. The integral of this Berry curvature yields a Chern number +1, thus demonstrating the topological nature of the band gap above. In contrast, for a trivial bandgap (e.g., one opened by inversion-symmetry breaking), the two peaks have opposite signs, so the resulting Chern number vanishes.

The total Chern number is conserved, and the nonzero Chern number of the band *i* results from the exchange with upper bands during the opening of the bandgap

by T-symmetry breaking. The exchange of Chern numbers involve three bands, because the two bands above the Dirac points (denoted as bands *ii* and *iii*) also have a degenerate point at the Γ point (see Supplementary Fig. 2a). Under a magnetic field, the Chern numbers of the bands *ii* and *iii* are 0 and -1, respectively (see Supplementary Fig. 2d,e), and therefore the sum of the Chern numbers of the three bands *i*-*iii* is conserved as zero.

The exchange of Chern numbers between the three bands can be understood from the distribution of Berry curvatures. The Chern number +1 gained by the band *i* is provided by the band *ii*, as clearly seen from the two peaks with negative signs in the Berry curvature of band *ii* at the K and K' points. However, the Chern number of band *ii* is zero, because the it also exchanges Chern number with band *iii* at the Γ point, as shown by the bending of the Berry curvatures of both bands *ii* and *iii* at the corner of the first Brillouin zone. Consequently, the Chern number of band *iii* is -1. Therefore, the total Chern number of the three bands *i*-*iii* is conserved.

Supplementary Note 3. Electrostatic scaling of graphene plasmons

The general electrostatic potential associated with a plasmon is $\phi(\mathbf{r},\omega)=\left(4\pi\varepsilon_0\bar{\varepsilon}\right)^{-1}\int d^2\mathbf{r}'\left|\mathbf{r}'-\mathbf{r}\right|^{-1}\rho(\mathbf{r}',\omega)$, where the $\rho(\mathbf{r}',\omega)$ is the charge distribution on the graphene, which is related to the surface current through the continuity equation, $\nabla\cdot\mathbf{j}(\mathbf{r}',\omega)=-i\omega\rho(\mathbf{r}',\omega)$, while $\bar{\varepsilon}=\left(\varepsilon_\mathrm{s}+\varepsilon_\mathrm{m}\right)/2$ is the average permittivity of the

environment (substrate $\varepsilon_{\rm s}$ and superstrate $\varepsilon_{\rm m}$) and a substrate. The current is $\mathbf{j}(\mathbf{r}',\omega)=\boldsymbol{\sigma}\cdot\mathbf{E}(\mathbf{r}',\omega)=\boldsymbol{\sigma}\cdot\nabla\phi(\mathbf{r}',\omega)$. Therefore, using a dimensionless coordinate vector $\boldsymbol{\theta}=\mathbf{r}/W$, the electrostatic potential can be expressed as

$$\phi=\frac{1}{4\pi\varepsilon_0\bar{\varepsilon}}\left[\eta_1\int\frac{d^2\boldsymbol{\theta}'}{|\boldsymbol{\theta}'-\boldsymbol{\theta}|}\left(\partial_x\phi+\partial_y\phi\right)+\eta_2\int\frac{d^2\boldsymbol{\theta}'}{|\boldsymbol{\theta}'-\boldsymbol{\theta}|}\left(\partial_y\phi-\partial_x\phi\right)\right],$$

where $\eta_1=i\sigma_{xx}/\omega W$ and $\eta_2=i\sigma_{xy}/\omega W$ are functions of the normalized frequencies ω/ω_0 and $\omega_{\rm c}/\omega_0$, where $\omega_0=\sqrt{e^2E_{\rm F}/\left(W\varepsilon_0\bar{\varepsilon}h^3\right)}$.

The above analysis shows that the plasmon fields in the graphene superlattices under study only depend on the geometrical parameters W and L, and on the normalized frequencies ω/ω_0 and $\omega_{\rm c}/\omega_0$. We provide a numerical corroboration of this electrostatic scaling law in Supplementary Fig. 3. These simulations demonstrate that sub-tesla magnetic fields are sufficient to generate topologically protected plasmon states. By carrying out FEM simulations similar to those used to obtain Fig. 2a, in which we calculate the scattering coefficients of a single nanoribbon junction (with L=3W, similar to Figs. 2-5), but with different combinations of parameters as a function of ω/ω_0, with W adjusted to ensure that $\omega_{\rm c}/\omega_0$=0.31 is same as in Fig. 4d. The simulation results show that the scaling law is perfectly fulfilled for different ribbon widths, doping levels, substrate environments and strengths of the magnetic field. These results also indicate that a superlattice of

larger size (W=806 nm with L=3W) operating under a magnetic field of only 0.8 T and a plasmon energy of 14 meV exactly resembles the results of Fig. 4d. Therefore, the required magnetic field can be further decreased into sub-tesla regime when the lower-energy plasmons are considered.

Supplementary References

[1] Wang, W., Apell, P. & Kinaret, J. Edge plasmons in graphene nanostructures. *Phys. Rev. B* **84**, 085423 (2011).

[2] Mast, D. B., Dahm, A. J. & Fetter, A. L. Observation of bulk and edge magnetoplasmons in a two-dimensional electron fluid. *Phys. Rev. Lett.* 54, 1706-1709 (1985).

[3] Xia, X. & Quinn, J. J. Edge magnetoplasmons of two-dimensional electron-gas systems. *Phys. Rev. B*, **50**, 11187-11189 (1994).

[4] Wang, W., Kinaret, J. M., & Apell, S. P. Excitation of edge magnetoplasmons in semi-infinite graphene sheets: Temperature effects. *Phys. Rev. B* **85**, 235444 (2012).

[5] Wang, W., Apell, S. P. & Kinaret, J. M. Edge magnetoplasmons and the optical excitations in graphene disks. *Phys. Rev. B* **86**, 125450 (2012).
